# SO_2_ gas adsorption on carbon nanomaterials: a comparative study

**DOI:** 10.3762/bjnano.9.169

**Published:** 2018-06-13

**Authors:** Deepu J Babu, Divya Puthusseri, Frank G Kühl, Sherif Okeil, Michael Bruns, Manfred Hampe, Jörg J Schneider

**Affiliations:** 1 Fachbereich Chemie, Eduard-Zintl-Institut für Anorganische und Physikalische Chemie, Alarich-Weiss-Strasse 12, Technische Universität Darmstadt, 64287 Darmstadt, Germany; 2 Fachgebiet Thermische Verfahrenstechnik, Otto-Berndt-Straße 2, Technische Universität Darmstadt, 64287 Darmstadt, Germany; 3 Institute for Applied Materials (IAM-ESS) and Karlsruhe Nano Micro Facility (KNMF), Hermann-von-Helmholtz-Platz 1, Karlsruhe Institute of Technology (KIT), 76344 Eggenstein-Leopoldshafen, Germany

**Keywords:** adsorption, carbon nanohorns, carbon nanotubes, heat of adsorption, sulfur dioxide, vertically aligned carbon nanotubes

## Abstract

Owing to their high stability against corrosive gases, carbon-based adsorbents are preferentially used for the adsorptive removal of SO_2_. In the present study, SO_2_ adsorption on different carbon nanomaterials namely carbon nanohorns (CNHs), multiwalled carbon nanotubes (MWNTs), single-walled carbon nanotubes (SWNTs) and vertically aligned carbon nanotubes (VACNTs) are investigated and compared against the adsorption characteristics of activated carbon and graphene oxide (GO). A comprehensive overview of the adsorption behavior of this family of carbon adsorbents is given for the first time. The relative influence of surface area and functional groups on the SO_2_ adsorption characteristics is discussed. The isosteric heat of adsorption values are calculated to quantify the nature of the interaction between the SO_2_ molecule and the adsorbent. Most importantly, while chemisorption is found to dominate the adsorption behavior in activated carbon, SO_2_ adsorption on carbon nanomaterials occurs by a physisorption mechanism.

## Introduction

Compared to the conventional techniques such as absorption in liquids, the adsorptive removal of environmentally toxic gases, e.g., SO_2_, offers several advantages such as ease of regeneration, low maintenance and simple plant design [1–2]. Consequently, in the last few years a wide variety of adsorbents has been investigated, e.g., for flue gas scrubbing applications. These include, but are not limited to, various zeolites [3–4], metal-organic frameworks [5–8], mesoporous silica [9–11] and carbon nanomaterials [12–15]. Due to its higher stability against moisture and corrosive gases (typical flue gas conditions), carbon-based adsorbents are particularly interesting for SO_2_ removal. In fact, activated carbon materials are one of the most widely used sorbents for SO_2_ recovery [1]. Over the past two decades, a rich family of different carbon nanomaterials such as fullerenes, carbon nanotubes (CNTs), carbon nanohorns (CNHs), graphene and graphene oxide were discovered. Unlike activated carbon, these nanomaterials have a defined geometry with distinct pore structure. Sun et al. investigated the SO_2_ adsorption characteristics of SWNTs, MWNTs and activated carbon at atmospheric pressure and at very low SO_2_ concentrations [16]. Here, a comparative study of the SO_2_ adsorption characteristics of a wider array of carbon nanomaterials like CNT, VACNTs, CNHs and GO is carried out in pure SO_2_ atmosphere at various temperatures up to its saturation pressure.

**Figure 1:**
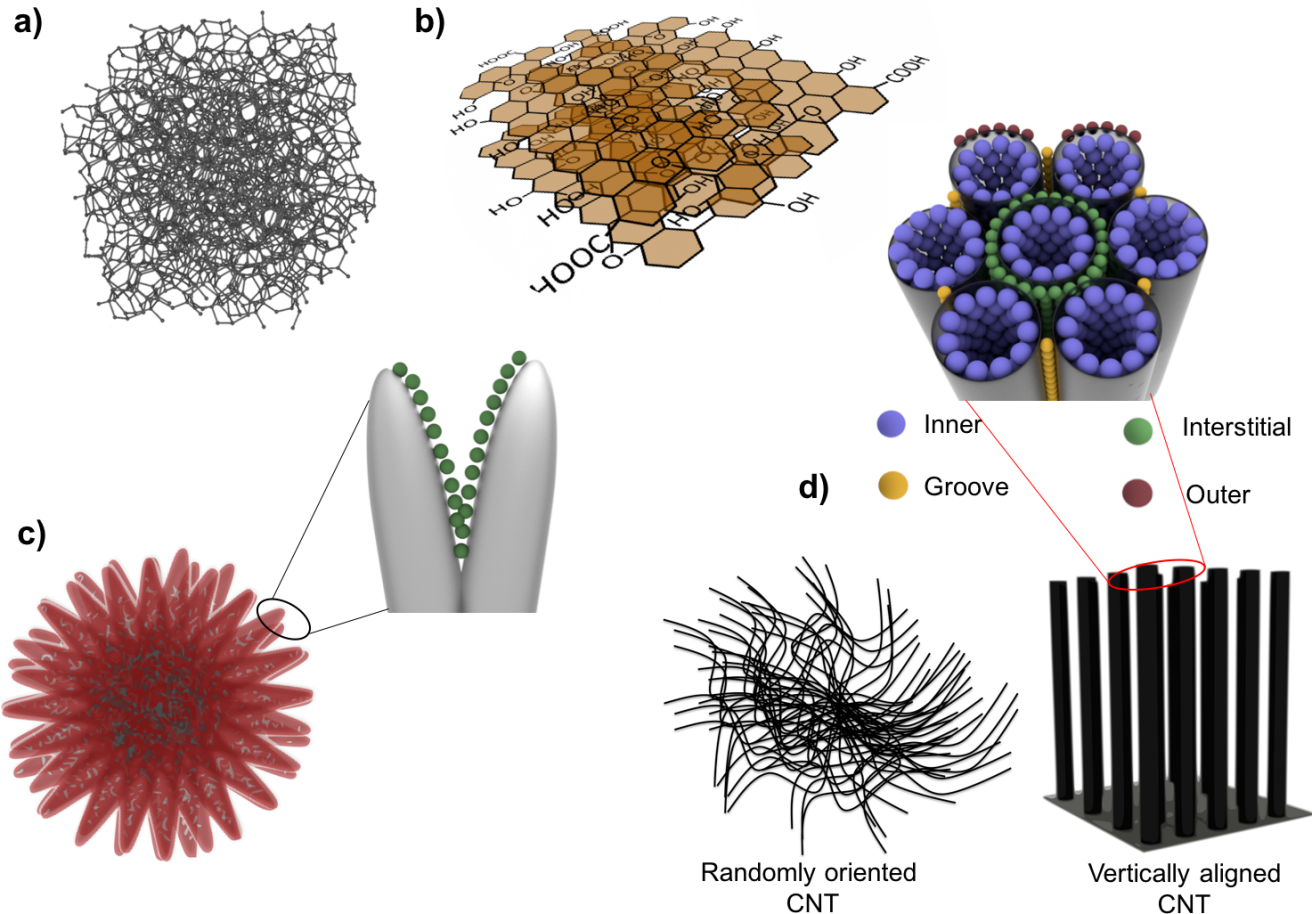
Schematic of different adsorbents: a) activated carbon, b) graphene oxide, GO (for the ease of presentation the carbon skeleton in b) is drawn in a planar fashion; however, there is noticeable deviation from planarity in GO); c) carbon nanohorns, d) carbon nanotubes.

A schematic of the different adsorbents investigated in this work is shown in Figure 1. Activated carbon, Norit R1 Extra, has an unordered pore structure (Figure 1a) and the porosity arises from the random stacking of the basic structural unit, which may be planar aromatic structures of less than 10–20 rings extending over 2–4 layers [17] or defective micro-graphene layers [18]. With this morphology it represents a typological carbon adsorbent with extended structural disorder. Graphene oxide (GO) has a 2D layered structure as shown schematically in Figure 1b. The starting material for the synthesis of GO is graphite, the oxidation of which introduces oxygen functionalities, which in turn increases the layer separation and turns the material hydrophilic. The subsequent exfoliation step separates the layers, thereby significantly increasing its accessible surface area leading to the formation of single- or few-layered GO. In our previous work, by XPS analysis, we have shown that the oxygen functionalities present on GO are in the form of hydroxy and carboxy groups [12]. The tunability of the material in terms of porosity and extent of functionalization makes GO a prototype of a hydrophilic carbon adsorbent and as such interesting for studying gas adsorption in 2D materials. Carbon nanohorns (CNHs) have a tubular structure with a closed cone-tip structure at one end (Figure 1c). Individual CNHs are usually single-walled with an internal diameter of 2–4 nm. The unique characteristic of CNHs is the rigid spherically aggregated structure with diameters of 50–100 nm [19–20]. The as-synthesized CNHs are closed and the interior of the CNHs is inaccessible for the gas molecules. An oxidative treatment such as heating in air, O_2_ [21–22] or CO_2_ [23–24] is used to open CNHs, thereby increasing the accessible surface area 3–4-fold. CNHs have a combination of micro–mesopores and are interesting for gas adsorption applications as they can be produced in large quantities with high purity [25–26].

CNTs have a well-defined structure as well and can be envisioned as a seamlessly rolled up graphene sheet. Since in a SWNT, the inside and the outside surfaces are available for adsorption, the theoretical surface area is in excess of 2500 m^2^/g [27]. However, in practice the surface area is much lower as CNTs seldom exist as isolated SWNTs but typically aggregate to form bundles that reduce the available surface area significantly. Depending on the synthesis method, the CNTs can be either randomly oriented or arranged parallel to each other resulting in a preferential alignment (Figure 1d). By the proper choice of synthesis parameters, the CNT growth orientation perpendicular to the substrate can be realized and such 3D CNT structures are referred to as vertically aligned CNTs (VACNTs). Compared to randomly oriented CNTs, VACNTs preserve the characteristic bundled morphology over macro-sized dimensions leading to a well-defined structure as shown in Figure 1d. Gases can adsorb on the interior of the CNTs known as the endohedral sites, on interstitial sites formed due to the parallel stacking on CNTs in such VACNT bundles, on the groove sites present at the intersection of two CNTs, as well as on the outer periphery [28]. These different adsorption sites vary in their adsorption energies due to the difference in the coordination number of carbon atoms and curvature effects [28–29]. The presence of these multiple well-defined and reproducible adsorption sites makes VACNTs an ideal model structure for investigating and understanding gas adsorption in one-dimensional carbon materials. In our previous works, we have shown the successful application of VACNTs as a model structure for a combined theoretical and experimental investigation of gas adsorption in carbon materials [30–31].

In the present study, the SO_2_ adsorption characteristics of the different carbon nanomaterials namely single-walled carbon nanotubes (SWNTs), multiwalled carbon nanotubes (MWNTs), vertically aligned carbon nanotubes (VACNTs) and carbon nanohorns (CNHs) are investigated and compared against the SO_2_ adsorption on activated carbon Norit R1 Extra and graphene oxide (GO). As the presence of oxygen and moisture (typical flue gas conditions) can complicate the interpretation of adsorption behavior, adsorption isotherms are recorded under pure SO_2_ atmosphere. The possibility to obtain pure SO_2_ gas equilibrium adsorption data under these experimental conditions is of great significance for theoretical adsorption investigations and gas mixture selectivity studies. For all the materials, the adsorption isotherms are measured at near ambient temperatures up to the saturation pressure.

## Results and Discussion

**Figure 2:**
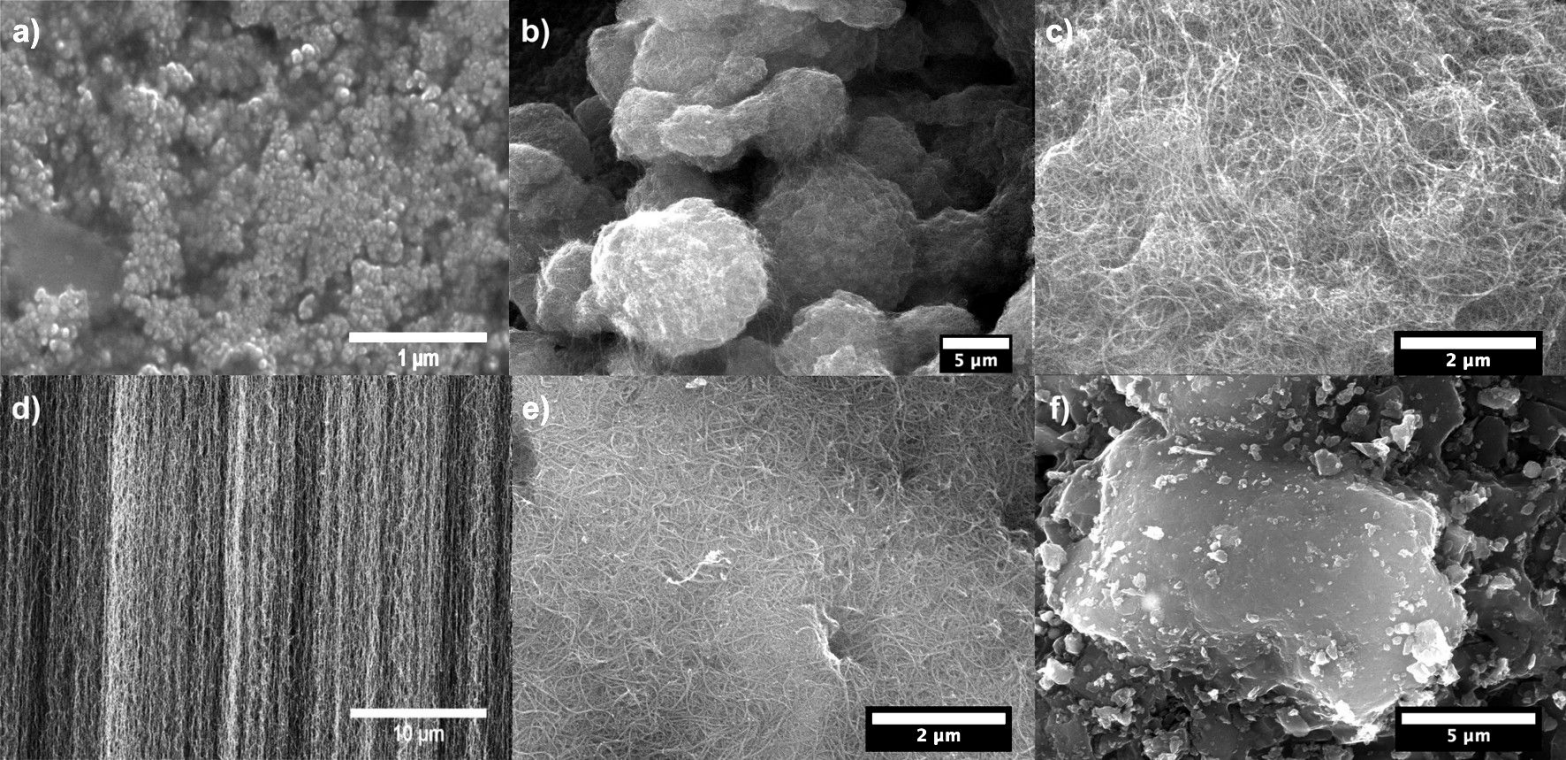
SEM image of the carbon samples used in the study: a) CNHs, b) MWNTs, c) high-magnification image of MWNTs revealing their unordered, felt-like structure, d) VACNTs revealing their alignment, e) unordered SWNTs, f) Norit R1 Extra.

**Figure 3:**
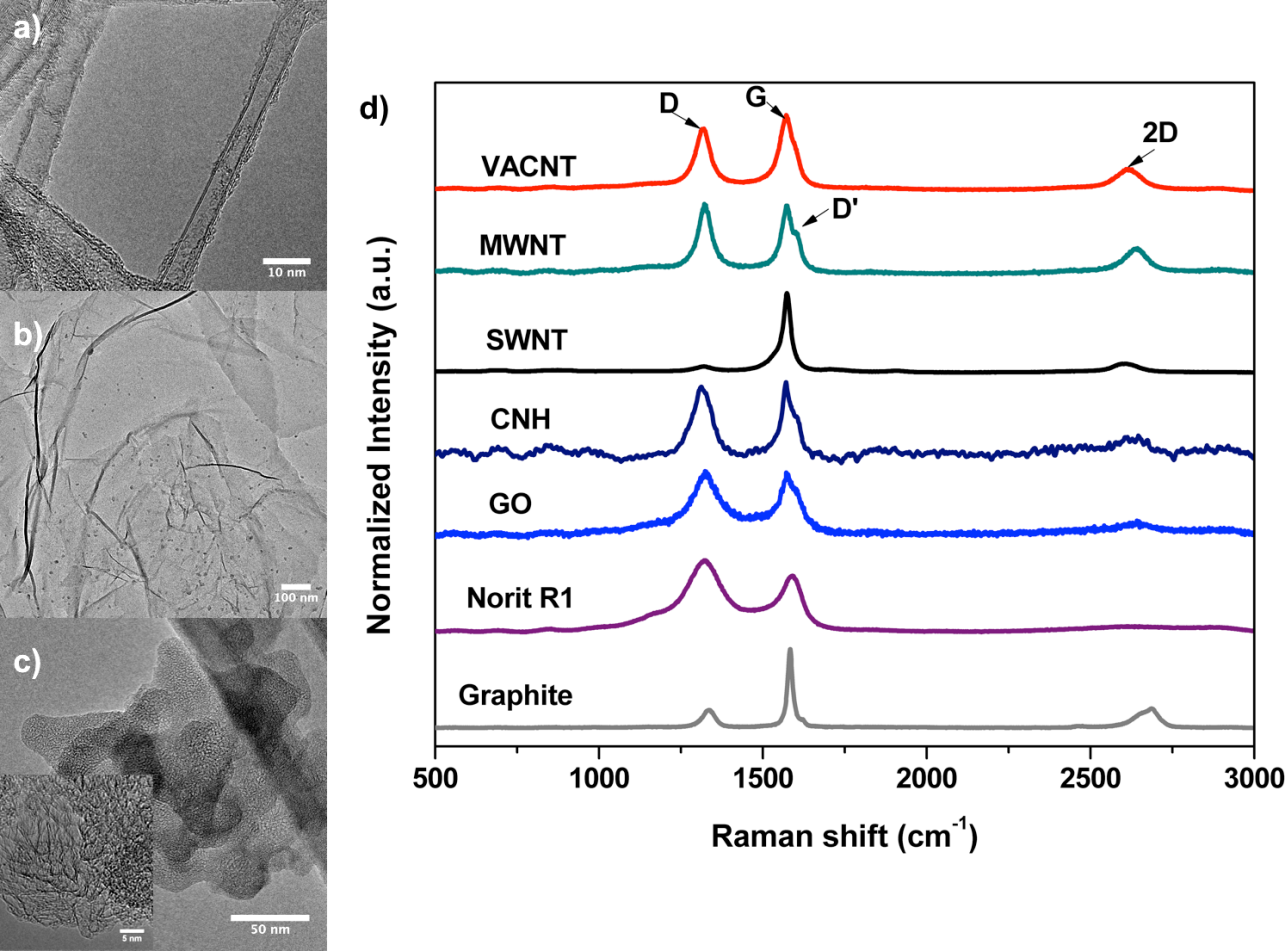
a) TEM image of VACNTs obtained after unhinging from the substrate and dispersed in ethanol by ultrasonication. b) TEM image of GO showing its characteristic wrinkled, layer like morphology c) TEM image of bud-like CNHs. Inset shows the high-magnification TEM image revealing the conical tips of CNHs. d) Overview of Raman spectra of the different adsorbents studied.

The morphology of the different adsorbents investigated in this work determined using SEM is shown in Figure 2. CNHs have an aggregated structure as shown by the SEM image in Figure 2a. TEM images of CNHs (Figure 3c) reveal only few tips that are protruding out of the spherical aggregate structure indicating a bud-like CNH structure [19–20]. The high-resolution TEM images confirm the single-walled conical structure of CNHs (inset of Figure 3c). The diameter of the individual CNHs is estimated to be between 1 and 4 nm. MWNTs exist in the form of bundled aggregates (ball-shaped, Figure 2b). High-magnification SEM images (Figure 2c) show that the MWNTs are randomly oriented. The SEM image of VACNTs (Figure 2d) demonstrates the preferential orientation of the CNTs. The average height of the VACNT structure obtained after 15 min of synthesis is ca. 800 µm. TEM investigations on numerous batches of as-synthesized VACNTs indicate double- to few-walled (number of walls ≤ 5) CNT structures with an average diameter of about 8 nm (Figure 3a). Like MWNTs, SWNTs grown by the CVD process have a randomly oriented structure (Figure 2e). The average diameter of the SWNTs and MWNTs are about 1.5 nm and 15 ± 5 nm, respectively (NanoLab, Inc. MA, USA). The commercially available activated carbon Norit R1 Extra is in pelletized form and the SEM image (Figure 2f) reveals an aggregated structure with no particular structure/ordering, which is characteristic for activated carbon materials. The pores of Norit R1 Extra are too small to be resolved by SEM.

In the following, an overview of the Raman characteristics of all studied carbon materials is given, which is followed by porosity evaluation by N_2_ adsorption isotherm measurement and a detailed XPS analysis of their surface functionalities. Finally, the gas adsorption studies of all materials are presented and compared comprehensively.

Raman spectroscopy is one of the few meaningful characterization techniques that are able to distinguish between various carbon materials containing sp^2^-hybridized carbon atoms. Raman spectra of the different adsorbents investigated in this work are given in Figure 3d. For reference, the Raman spectrum of graphite is also given. The G-band or graphite band (ca. 1585 cm^−1^) is the Raman signature for all sp^2^-hybridized carbon materials and arises from the in-plane stretching mode of the C–C bond [32]. The D-band (ca. 1350 cm^−1^) is a defect-activated vibrational mode where the defects act as an elastic scattering center to assist the intervalley double-resonance process. Its intensity is proportional to the presence of defects or disorder in the material and is strongly dependent on the laser excitation energy [32]. The 2D band for sp^2^-hybridized carbon materials is found between 2500 and 2800 cm^−1^ and is a double-resonance two-phonon process [33]. The 2D band intensity is found to be inversely proportional to the concentration of defects in the structure [34]. The D′-band (ca. 1620 cm^−1^) is also another defect-induced band which is assigned to the in-plane vibrations of the outer parts of the graphite domains [35–36]. It is typically observed for MWCNTs and intercalated graphite compounds [36]. The intensity ratios of D- to G-band (*I*_D_/*I*_G_) calculated for the different adsorbents are given in Table 1. Graphite and SWNTs have a very low D-band intensity indicating fewer defects in the structure. The Raman spectrum of SWNTs also indicates the presence of a characteristic RBM mode (Figure S1 in Supporting Information File 1). The Raman spectrum for CNHs is characterized by similar intensities of the D- and G-bands where the D-band intensity is mainly due to the presence of the pentagon rings in the cone region of individual CNHs [24,37]. In GO, similar to CNHs, the D-band and G-band are equally intense and is an indicator of the structural distortion induced by the attachment of a large number of functional groups [12,38]. The D- to G-band intensity ratios for VACNTs and MWNTs are 0.83 and 1.02, respectively. From our TEM investigations we could corroborate that the as-synthesized VACNTs are seldom straight with a uniform diameter and the diameter varies along the axis due to the presence of defects (Figure S2 in Supporting Information File 1). The presence of such defects is known to contribute to an increase in the D-band intensity [39]. The Raman spectra of MWNTs and VACNTs also indicate the presence of a D′-band due to vibrations from the outer CNT walls. The Raman spectrum of activated carbon Norit R1 Extra is characterized by a broad D-band indicating the presence of different types of defects. Also notable is the near total absence of 2D band in Norit R1 Extra indicating maximum disorder in the structure.

**Table 1:** The intensity ratios of D-band to G-band calculated from the Raman spectra of the different adsorbents.

sample	*I*_D_/*I*_G_	sample	*I*_D_/*I*_G_

graphite	0.25	SWNT	0.09
Norit R1 Extra	1.24	MWNT	1.02
GO	1.02	VACNT	0.83
CNH	0.94		

**Figure 4:**
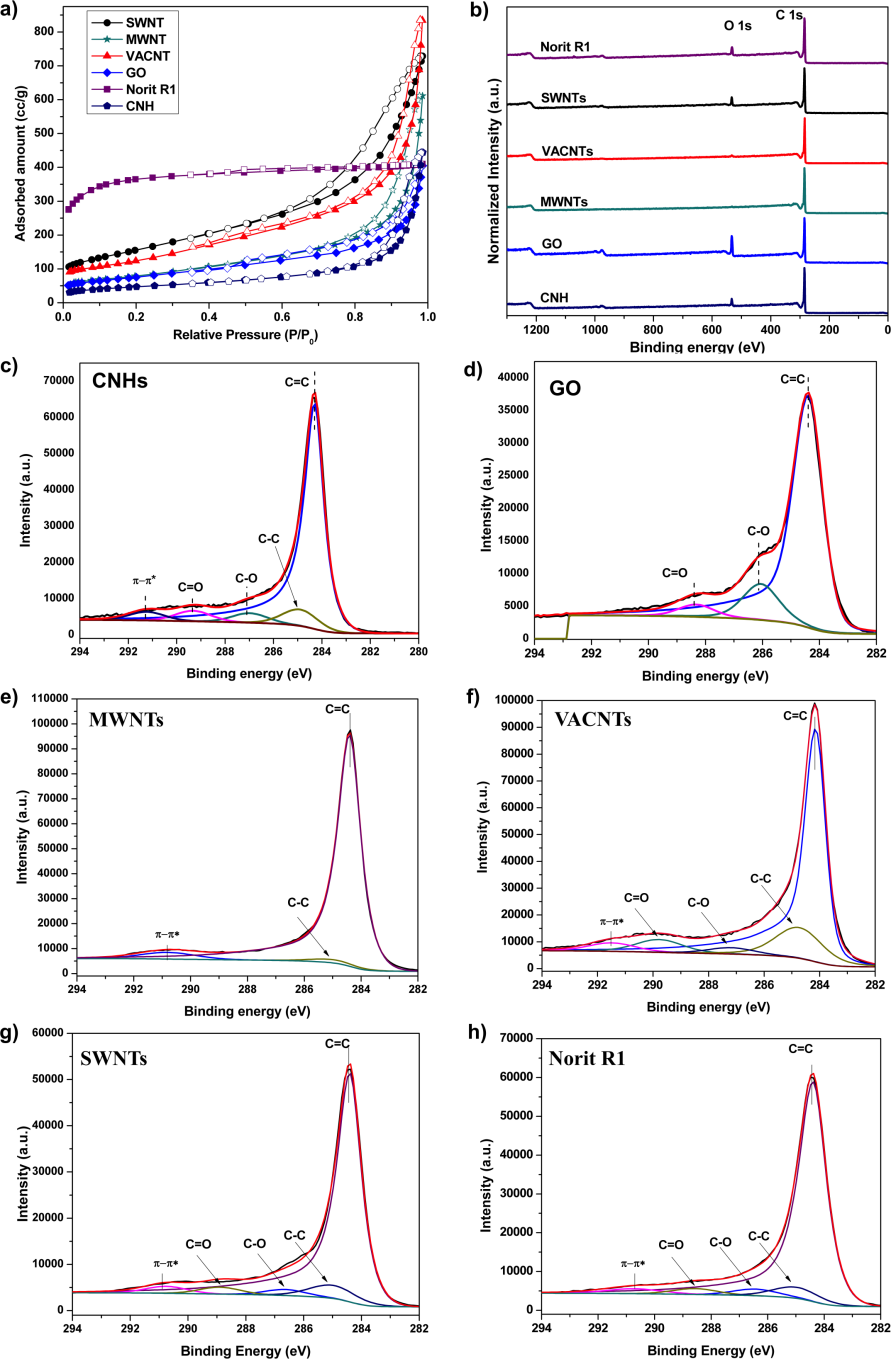
a) N_2_ adsorption isotherm at 77 K and b) survey spectra of the six different carbon adsorbents studied. C 1s spectrum of c) CNHs, d) GO [12] e) MWNTs, f) VACNTs, g) SWNTs and h) Norit R1 Extra.

The N_2_ adsorption isotherms at 77 K of the six adsorbents are plotted in Figure 4a. The predominantly microporous nature of Norit R1 Extra is evident from the Langmuir-type adsorption isotherm (type I). The observed steep adsorption at low relative pressures is due to enhanced adsorptive–adsorbent interactions in the narrow micropores present in this material. The apparent surface area of Norit R1 Extra is estimated to be ca. 1375 m^2^/g and a *t*-plot analysis reveals the micropore contribution towards the total surface area to be about 89.5% (Table 2). Except for activated carbon Norit R1 Extra, all the adsorbents exhibit a type-IVa adsorption isotherm according to the revised IUPAC classification [40]. Among the different adsorbents investigated in the present study, CNHs have the lowest surface area (Table 2). The as-synthesized CNHs are usually closed and the hollow inner core of the CNHs is inaccessible for the adsorbent molecules [21,41]. For CNTs, it is known that the surface area decreases with an increase in the diameter of the individual tubes and the number of walls. A similar trend is observed in the present study with SWNTs exhibiting the highest accessible surface area of 557 m^2^/g, followed by VACNT (438 m^2^/g) and MWNTs (284 m^2^/g).

**Table 2:** BET surface area and *t*-plot analysis of the different adsorbents.

sample	BET surface area (m^2^/g)	*t*-plot method	
		micropore area (m^2^/g)	external surface area (m^2^/g)

Norit R1 Extra	1375	1230	145
GO	268	4	264
CNH	168	22	146
SWNT	557	2.9	554
MWNT	284	0	283
VACNT	438	0	438

Adsorption is a function of not only the pore structure and geometry but also of the chemical composition. To fully understand the adsorption behavior of an adsorbent it is imperative to characterize its chemical composition as well. Due to their high absorption near the infrared region, IR spectroscopy is seldom used to characterize CNTs and CNHs. In contrast, XPS is a central characterization method that can be successfully applied to different types of carbon materials to obtain meaningful chemical information on surface functionalities. In Figure 4b, the XPS survey spectra of the different adsorbents are shown. Among the six adsorbents, CNHs, GO, SWNTs and Norit R1 Extra display a significant oxygen concentration on their surface. Influence of oxygen functional groups especially carbonyl groups, on SO_2_ physisorption was theoretically studied by Furmaniak and co-workers [42]. Using the hyper-parallel tempering Monte Carlo method, they found that the influence of the oxygen functionalities is more pronounced at lower relative pressures (*P*/*P*_0_ < 0.3) and attributed it to the increase in the adsorption energy caused by the electrostatic interactions of SO_2_ molecules with CO functionalities. By investigating the adsorption of a multicomponent mixture containing SO_2_, NO, chlorobenzene and H_2_O on activated carbon using a fixed-bed reactor, Li et al. observed that the presence of carbonyl groups enhances the SO_2_ adsorption [43]. By combining in situ powder X-ray diffraction and inelastic neutron scattering measurements with simulation studies, Yang et al. argued that hydroxy groups within the pore channels selectively bind SO_2_ by the formation of hydrogen bonds that are reinforced by weaker phenyl C–H^…^O=S=O supramolecular contacts surrounding the pore [2]. The beneficial role of hydroxy groups on SO_2_ adsorption was also observed in the case of flue gas adsorption on MOFs [8]. All these studies point to the fact that the presence of oxygen functionalities can certainly influence the SO_2_ adsorption characteristics of an adsorbent. In the present study, the deconvoluted high-resolution scans reveal that GO has the highest oxygen concentration (16.1 atom %) followed by Norit R1 Extra (8.1 atom %) (Figure 4c–h and Table S1 in Supporting Information File 1). The VACNTs and MWNTs have negligible amount of oxygen functionalities on the surface (1.43 atom % for VACNTs). For VACNTs, using XPS depth profile measurements we have previously shown that the oxygen functionalities are present only in the top few nanometers of typically several hundred micrometer long CNTs [44]. The results of XPS quantitative analysis are summarized in Table S1 (Supporting Information File 1) and the detailed C 1s deconvolution is given in Table S2 (Supporting Information File 1).

**Figure 5:**
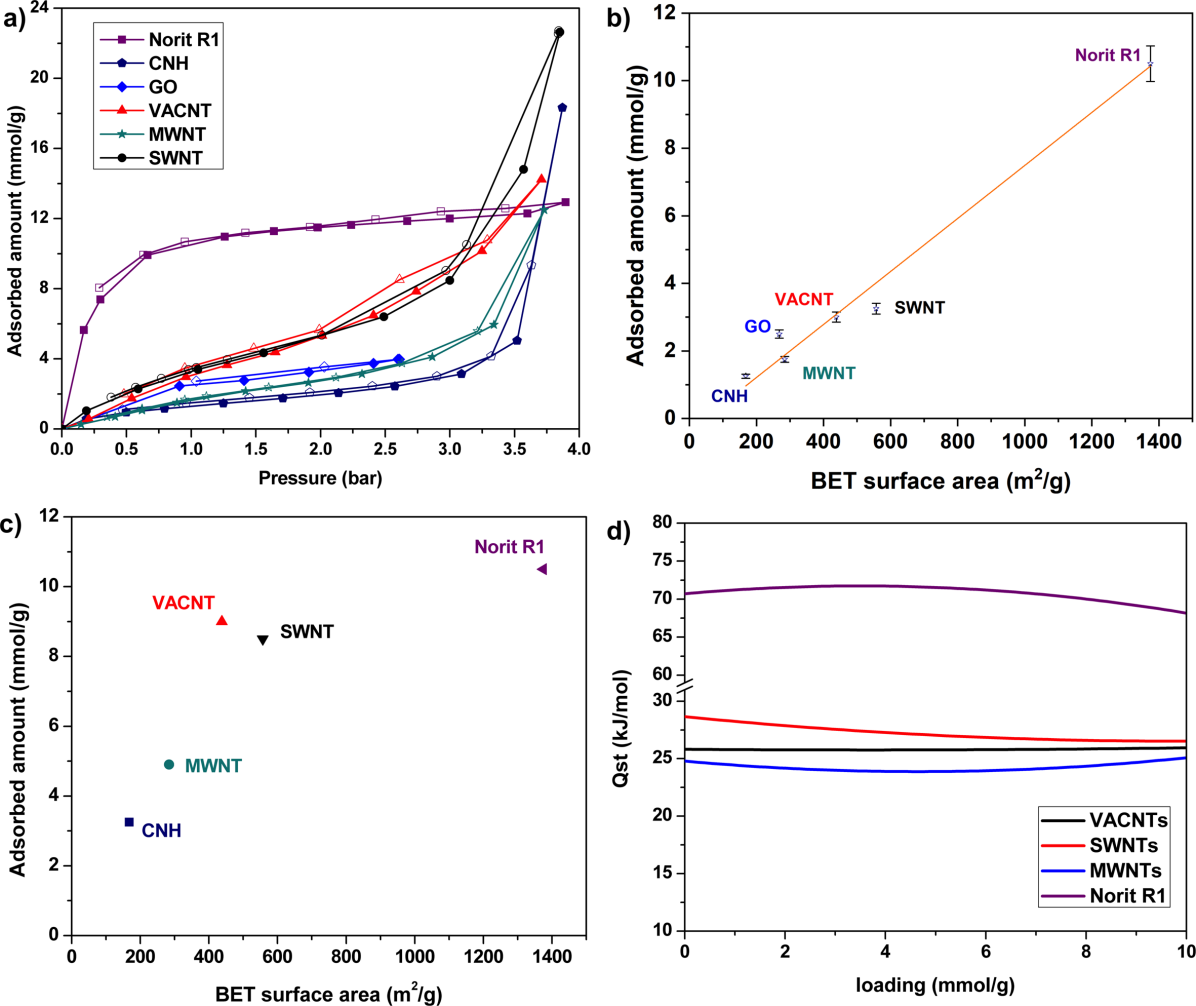
a) SO_2_ adsorption isotherms at 25 °C of different adsorbents studied up to the saturation pressure of SO_2_. The results of GO are from [12]. SO_2_ adsorption as a function of BET specific surface area at b) 1 bar and at c) 3 bar. d) Heat of adsorption of carbon nanotubes and Norit R1 Extra.

The SO_2_ adsorption isotherm of the six adsorbents at 25 °C is shown in Figure 5a. The experimental measurement errors are ±5% for the loading and the pressure reading. It should be noted that at higher relative pressures (*P*/*P*_0_ > 0.7), the condensation effect leads to larger deviations and greater error. This is because, in this range, as the density of the gas approaches the density of the liquid, minor deviations in temperature result in large variations of the adsorbed amount of gas. Nevertheless, an analysis shows an error of below 5% depending on the calculated density. The errors are related to the systematic error regarding the measured values of temperature and pressure. An effect of the sample cannot be considered. Detailed information can be found in [45]. Under near-ambient conditions, activated carbon Norit R1 Extra exhibits the highest SO_2_ adsorption capacity. The significantly higher SO_2_ adsorption capacity for activated carbon compared to other adsorbents can be attributed to the presence of micropores. As indicated by the *t*-plot analysis, micropores constitute almost 89.5% of the total available surface area of activated carbon Norit R1 Extra. At 1 bar, CNHs have the lowest intake of SO_2_ followed by MWNTs and GO. In Figure 5b, the adsorbed amount of SO_2_ at 1 bar is plotted as a function of the BET specific surface area of the adsorbent. At 1 bar, there is an almost linear relationship between the BET specific surface area and the SO_2_ adsorption. The notable exceptions are GO and SWNT. The high SO_2_ uptake by GO is presumed to be due to the high concentration of oxygen functionalities (16.1 atom %) that are easily accessible due to its layered structure. Consistent with the theoretical predictions of Furmaniak et al. [42], the influence of oxygen functionalities is found to be more pronounced under near-ambient conditions where the relative pressure *P*/*P*_0_ is less than 0.3. In our TEM measurements of SWNTs, we have seen the presence of few MWNTs and other catalyst impurities. The presence of these foreign particles might be one of the reasons for the observed low SO_2_ uptake in SWNTs. In Figure 5c, the SO_2_ adsorption at 3 bar is plotted as a function of the specific surface area of the adsorbent. At this pressure, CNHs, MWNTs and SWNTs lie on the same line while VACNTs exhibits a higher SO_2_ adsorption capacity. The SO_2_ uptake of Norit R1 Extra is no longer in line with the specific surface at a pressure of 3 bar. This is because, unlike other adsorbents, all the microporous adsorption sites of Norit R1 Extra are already filled at nearly 1 bar and further increase in pressure leads to only very little SO_2_ uptake. The higher adsorption capacity of VACNTs can be explained by the presence of multiple adsorption sites in VACNTs. Although SWNTs and MWNTs exist in the form of bundles, which in principle leads to multiple adsorption sites, in VACNTs the alignment ensures that the bundled morphology extends over macroscopic dimensions. Simulation studies of Yang et al. on double-walled aligned CNTs have shown that the outer adsorption sites (interstitial + groove + outside periphery) constitute a significant fraction (>50%) of the total amount of SO_2_ adsorbed [46]. The higher SO_2_ uptake observed for VACNTs is consistent with the reports of enhanced N_2_ and H_2_ uptake observed for aligned CNTs [47–49].

The heat of adsorption gives a quantitative estimate of the interaction between the adsorbate and the adsorbent. In the present study, the isosteric heat of adsorption was calculated from the isotherms measured at 15, 25 and 35 °C according to the method of Czepirski and co-workers [50] and Sun co-workers [51]. Briefly, the isotherms at the three different temperatures were fitted to the equation:


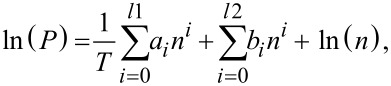


where *n* is the adsorbed amount at pressure *P* and temperature *T*; *a* and *b* are empirical parameters. Subsequently the isosteric heat of adsorption was calculated according to the relation:


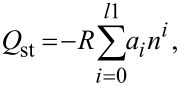


where *R* is the universal gas constant. For Norit R1 Extra, the heat of adsorption was calculated from the two isotherms at 25 and 35 °C. In our previous work on GO, the heat of adsorption of GO was determined to be 16.87 kJ/mol at a loading of 1 mmol/g [12]. In Figure 5d, the heat of adsorption of the three types of CNTs and Norit R1 Extra are shown. At a loading of 1 mmol/g, Norit R1 Extra has a heat of adsorption of ca. 71 kJ/mol. Physisorption alone cannot account for such a high value of the heat of adsorption. In activated carbons, it is known that the presence of oxygen functional groups and micropores in the 0.7 nm range can promote SO_2_ chemisorption [52–53]. The extensive microporous structure and the presence of oxygen functional groups in Norit R1 Extra explain this high heat of adsorption. Among the different types of CNTs, SWNTs exhibit the highest heat of adsorption ~28.2 kJ/mol at a loading of 1 mmol/g. The heat of adsorption on VACNTs lies between those of MWNTs and SWNTs. While SWNTs and MWNTs show an initial decrease in the heat of adsorption followed by an increase, VACNTs maintain an almost constant value of heat of adsorption. One reason for this behavior might be the presence of multiple adsorption sites in VACNTs. From the grand canonical Monte Carlo simulation studies of the SO_2_ adsorption on VACNTs, it is already known that depending upon the diameter and the intertube distance, the filling of a particular adsorption site can lead to either a decrease or an increase in the heat of adsorption with loading [46]. A conclusive answer, however, requires further detailed studies in this direction.

## Conclusion

In conclusion, the SO_2_ adsorption characteristics of different carbon nanomaterials were investigated and compared with an activated standard carbon adsorbent. Under near-ambient conditions, the activated carbon Norit R1 Extra exhibits significantly higher SO_2_ uptake than all the other adsorbents investigated in this work. The calculated isosteric heat of adsorption value of ca. 71 kJ/mol at a loading of 1 mmol/g suggests that SO_2_ adsorbs on Norit R1 carbon by a chemisorption mechanism. A comparison of the adsorption characteristics of the different adsorbents at 1 bar suggests a linear relationship of SO_2_ uptake with BET specific surface area. The presence of oxygen functionalities was found to favor SO_2_ adsorption and the influence was found to be more pronounced at lower relative pressures. The SWNTs, MWNTs and VACNTs adsorb SO_2_ by a physisorption process with heat of adsorption values between 25 and 30 kJ/mol. Even though the BET surface area differs by about 120 m^2^/g, VACNTs exhibit a similar SO_2_ uptake to that of SWNTs. The presence of multiple adsorption sites in VACNTs is assumed to be responsible for this observed enhanced adsorption.

## Experimental

### Materials

Activated carbon of type Norit R1 Extra was obtained from Norit N.V., Holland, and the detailed properties have been described in [45,54]. GO was prepared by oxidizing graphite followed by exfoliation using a combination of ultrasound sonication followed by freeze–thaw cycles, described in detail in [12]. Graphite was purchased from Fortune Graphite Inc., Canada (purity of 99.99%). The bud-like carbon nanohorn aggregates were of Type F, obtained from TIE GmbH, Griesheim, Germany. The CNHs were synthesized through arc discharge of graphite in water under inert atmosphere [55] and have a purity of >95% with no metal impurities present as characterized by TG, TEM, XPS and EDX (the remaining material being carbon materials such as fullerene fragments and graphite particles). SWNTs and MWNTs were obtained from NanoLab, Inc. MA, USA. According to the manufacturer, MWNTs have a purity of >95%, with an outer diameter of 15 ± 5 nm with a total length of 5–20 μm. SWNTs have a diameter of ca. 1.5 nm, a length of 1–5 µm and were produced by using CVD with a purity of >95%. Vertically aligned carbon nanotubes (VACNTs) were synthesized in our lab over a Si/SiO_2_ (600 nm) substrate in a quartz furnace using water-assisted chemical vapor deposition [56–57]. The bimetallic catalyst system for the VACNT growth was prepared by depositing a thin layer of aluminum (13–15 nm) over the substrate through thermal evaporation in a vacuum of 10^−6^ mbar, followed by the sputter deposition of 1.2 nm of an iron catalyst layer. The synthesis was carried out at 900 °C by passing ethene (200 sccm), hydrogen (800 sccm), argon (1200 sccm) and ppm-scale quantities of water vapor together for 15 min.

### Characterization techniques

Raman measurements were performed using a Horiba Jobin Yvon, model HR 800 LabRAM high-resolution microscope using a He–Ne laser with 632.8 nm as the excitation source. The instrument was calibrated to the silicon peak at 521 cm^−1^. Transmission electron microscopy (TEM) measurements were done using a FEI Tecnai F20 G2 operated at 200 kV. Scanning electron microscopy (SEM) images were recorded using Philips XL30 FEG operated at 20 kV. X-ray photoelectron spectroscopy (XPS) measurements were performed using a K-Alpha XPS spectrometer (ThermoFisher Scientific, East Grinstead, UK). Data acquisition and processing using the Thermo Avantage software is described elsewhere [58]. All samples were analyzed using a microfocused, monochromated Al Kα X-ray source (30–400 µm spot size). The K-Alpha charge compensation system was employed during analysis, using electrons of 8 eV energy and low-energy argon ions to prevent any localized charge build-up. The spectra were fitted with one or more Voigt profiles (binding energy uncertainty: ±0.2 eV). The analyzer transmission function, Scofield sensitivity factors [59] and effective attenuation lengths (EALs) for photoelectrons were applied for quantification. The EALs were calculated using the standard TPP-2M formalism [60]. All of the spectra were referenced to the C 1s peak of graphite at 284.4 eV binding energy controlled by means of the well-known photoelectron peaks of metallic Cu, Ag, and Au.

### Adsorption measurements

Nitrogen adsorption–desorption isotherms at 77 K were recorded using Quanta chrome 3000e instrument. Before the experiment, sample was degassed at 150 °C for 24 h for the removal of the moisture and other adsorbed gases on the sample. Analysis of the adsorption data were carried out with the software suite NOVA version 10. The BET surface area was calculated using the linearized form of multipoint BET. SO_2_ adsorption measurements were performed in a gravimetric setup IsoSORP Series SC-HP Static (Rubotherm, Bochum, Germany), in combination with an automatic gas-dosing system (in operation for blank and buoyancy measurements with helium and a manual gas dosing system, in operation for measurements with sulfur dioxide (both from Rubotherm, Bochum, Germany). The resolution of the used magnetic suspension balance was 1 µg. Experimental measurement errors were max. ±5% for the loading and pressure reading. Buoyancy effects of the floating parts of the setup and the sample were taken into account by helium correction measurements of the blank setup and the setup loaded with sample at the studied temperatures. The utilized helium was Alphagaz™ Helium supplied by Air liquide (purity 99.999%, N_2_ < 5 ppm_v_, O_2_ < 2 ppm_v_, H_2_O < 2 ppm_v_, HC < 0.2 ppm_v_ as stated by the supplier). Prior to each helium buoyancy correction, the sample was degassed at 150 °C for 12 h. Prior to blank measurements, the setup was degassed at 300 °C for 12 h. For SO_2_ adsorptions, SO_2_ with a purity of 99.98% was used (supplied by Air Liquide in the purity N38, H_2_O ≤ 50 ppm_w_, H_2_SO_4_ ≤ 10 ppm_w_ as stated by the supplier). Prior to SO_2_ measurements the sample was also degassed at 150 °C for 12 h under vacuum conditions better than 5 × 10^−2^ mbar. To achieve a sufficiently high back-pressure for SO_2_ for measuring till the saturation pressure at the respective temperature, SO_2_ was introduced into a syringe pump 500D from Teledyne ISCO equipped with a temperature control jacket and pressurized to 10 bar.

## Supporting Information

File 1 Additional experimental data.
