# Supplementary material for: SO2 gas adsorption on carbon nanomaterials: a comparative study
Source: Beilstein J Nanotechnol. 2018 Jun 13;9:1782–92. doi: 10.3762/bjnano.9.169 (PMC6009271; doi:10.3762/bjnano.9.169)
Supplement: File 1 — Additional experimental data. [file Beilstein_J_Nanotechnol-09-1782-s001.pdf]

# Supporting Information

for

# $SO_2$ gas adsorption on carbon nanomaterials: a comparative study

Deepu J. Babu‡[1], Divya Puthusseri‡[1], Frank G. Kühl‡[2], Sherif Okeil[1], Michael Bruns[3], Manfred Hampe[2] and Jörg J. Schneider*[1]

Address: [1]Fachbereich Chemie, Eduard-Zintl-Institut für Anorganische und Physikalische Chemie, Alarich-Weiss-Strasse 12, Technische Universität Darmstadt, 64287 Darmstadt, Germany; [2]Fachgebiet Thermische Verfahrenstechnik, Otto-Berndt-Straße 2, Technische Universität Darmstadt, 64287 Darmstadt, Germany and [3]Institute for Applied Materials (IAM-ESS) and Karlsruhe Nano Micro Facility (KNMF), Hermann-von-Helmholtz-Platz 1, Karlsruhe Institute of Technology (KIT), 76344 Eggenstein-Leopoldshafen, Germany

Email: Jörg J. Schneider* - joerg.schneider@ac.chemie.tu-darmstadt.de

* Corresponding author

‡ Equal contributors

## Additional experimental data

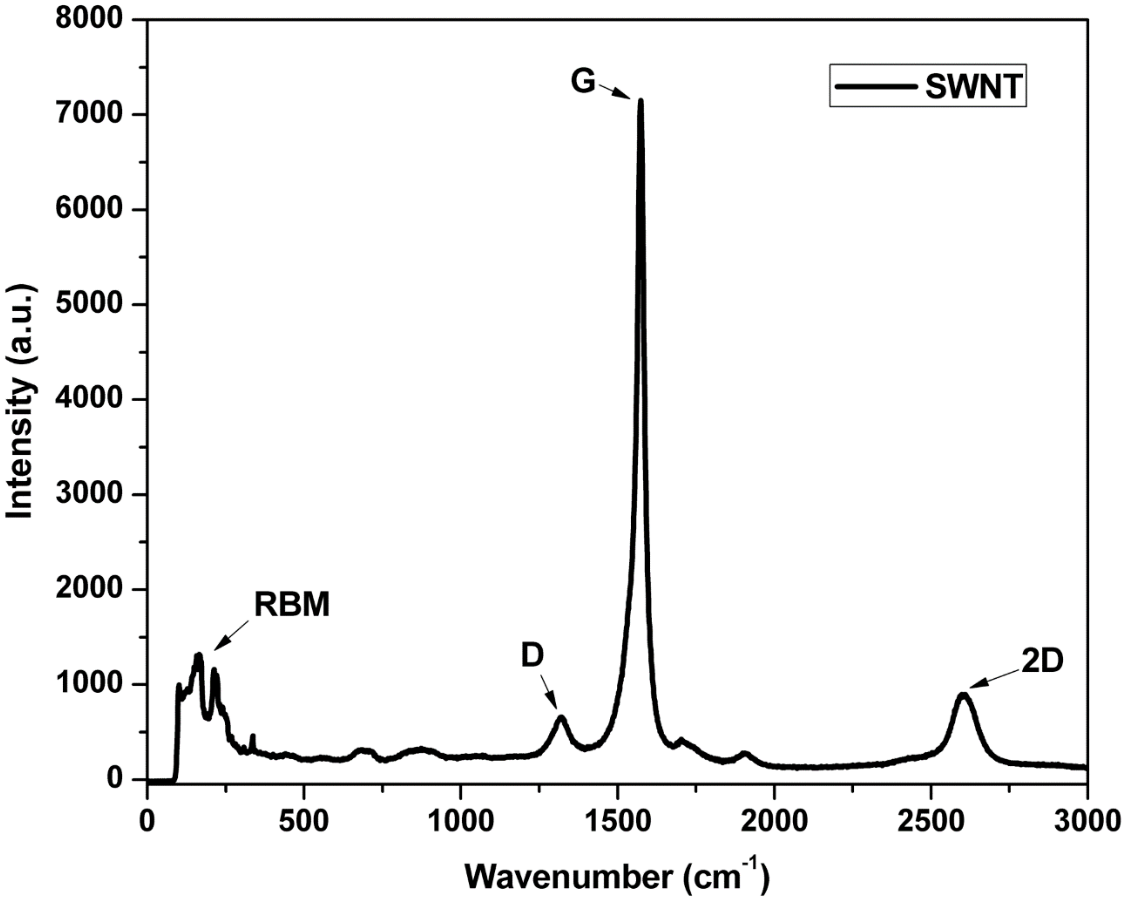


**Figure S1:** Raman spectrum of SWNTs showing the RBM mode.

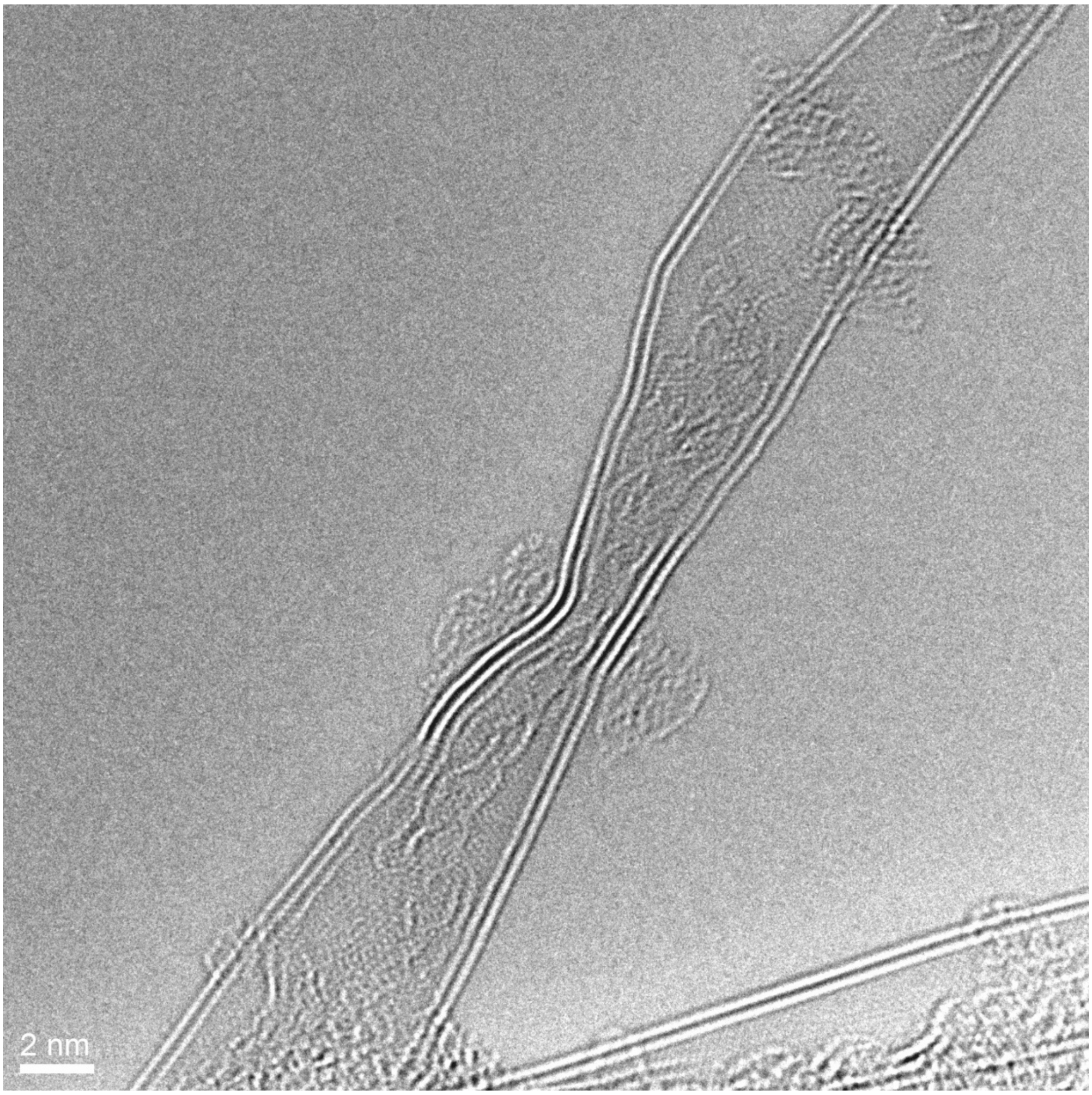


**Figure S2:** High-resolution TEM image of an isolated CNT unhinged from a VACNT array showing defects in the CNT and the accompanying variation of the diameter along the length of the CNT.

**Table S1:** XPS quantitative analysis of the different adsorbents.

| **sample** | **C (atom %)** | **N (atom %)** | **O (atom %)** |
|---|---|---|---|
| CNHs | 93.01 | 1.08 | 5.91 |
| GO | 83.9 | 0 | 16.1 |
| MWNTs | 100 | — | — |
| VACNTs | 98.57 | — | 1.43 |
| SWNTs | 92.3 | 1.2 | 6.5 |
| Norit R1 Extra | 91.9 | — | 8.1 |

**Table S2:** Deconvolution results of the XPS high-resolution C 1s scan of the different adsorbents.

| **sample** | **C (atom %)** | | | | |
|---|---|---|---|---|---|
| | C=C | C–C | C–O/C–N | C=O | π–π* |
| CNHs | 74.22 | 6.83 | 3.95 | 4.40 | 3.61 |
| GO | 68 | – | 10.5 | 4.1 | 1.3 |
| MWNTs | 93.3 | 1.9 | – | – | 4.8 |
| VACNTs | 68.88 | 15.58 | 3.24 | 6.53 | 4.34 |
| SWNTs | 78.1 | 6.1 | 2.4 | 2.8 | 2.9 |
| Norit R1 | 78.5 | 5.1 | 3.0 | 2.6 | 2.7 |
